# Adherence to cardiovascular medications and risk of cardiovascular disease in breast cancer patients: A causal inference approach in the Pathways Heart Study

**DOI:** 10.1371/journal.pone.0310531

**Published:** 2024-09-19

**Authors:** Marilyn L. Kwan, Noel Pimentel, Monika Izano, Carlos Iribarren, Jamal S. Rana, Mai Nguyen-Huynh, Richard Cheng, Cecile A. Laurent, Valerie S. Lee, Janise M. Roh, Eileen Rillamas-Sun, Dawn L. Hershman, Lawrence H. Kushi, Heather Greenlee, Romain Neugebauer

**Affiliations:** 1 Division of Research, Kaiser Permanente Northern California, Pleasanton, California, United States of America; 2 Insights Epidemiology and Analytics, Syapse, San Francisco, California, United States of America; 3 Cardiology, Oakland Medical Center, Kaiser Permanente Northern California, Oakland, California, United States of America; 4 Neurology, Walnut Creek Medical Center, Kaiser Permanente Northern California, Walnut Creek, California, United States of America; 5 Division of Cardiology, University of Washington Medical Center, Seattle, Washington, United States of America; 6 Division of Public Health Sciences, Fred Hutchinson Cancer Center, Seattle, Washington, United States of America; 7 Medical Oncology, Herbert Irving Comprehensive Cancer Center, Columbia University Irving Medical Center, New York, New York, United States of America; 8 Department of Health System Science, Kaiser Permanente Bernard J. Tyson School of Medicine, Pasadena, California, United States of America; Kampala International University - Western Campus, UGANDA

## Abstract

**Purpose:**

Women with breast cancer (BC) are at high risk of developing cardiovascular disease (CVD). We examined adherence to CVD medications and their association with major CVD events over 14 years of follow-up in the Pathways Heart Study, a prospective study of 4,776 stage I-III BC patients diagnosed from 2005–2013.

**Methods:**

Eligibility included being alive 6 months post-BC diagnosis, with dyslipidemia, hypertension, or diabetes at diagnosis along with ≥1 prior outpatient order or dispensing for a statin, anti-hypertensive, or diabetes medication, respectively, in the 30 months prior. Medication adherence was measured from pharmacy data to calculate cumulative average adherence (CAA). Incident heart failure (HF), ischemic heart disease (IHD), and stroke were determined via validated diagnosis and procedure codes. Working marginal structural models (MSM) fitted with inverse probability weighting evaluated the effect of adherence regimens on the hazards for each CVD event, while controlling for baseline and time-varying confounders. MSM parameterizations included: 1) CAA<100% versus CAA = 100% (ref), 2) CAA<80% versus CAA≥80% (ref) and 3) CAA<80% versus 80%≤CAA<100% versus CAA = 100%.

**Results:**

Poor statin adherence (CAA<80%) was associated with higher risk of composite CVD (HR = 2.54; 95% CI: 1.09, 5.94) versus CAA≥80%. Poor statin adherence was also associated with a higher risk of stroke (HR = 8.13; 95% CI: 2.03, 32.51) but not risk of IHD and HF. Further, compared with perfect adherence (CAA = 100%), good adherence (80%≤CAA<100%) was associated with lower risk (HR = 0.35; 95% CI: 0.13, 0.92) while poor adherence (CAA<80%) was associated with higher risk of composite CVD (HR = 2.45; 95% CI: 1.05, 5.70). Levels of adherence to anti-hypertensives and diabetes medications had mixed or null associations with risk of CVD.

**Conclusions:**

Maintaining good adherence (≥80%) to statins after BC treatment is beneficial for cardiovascular health in patients with dyslipidemia. Future studies should determine factors associated with lower adherence to statins and ways to improve adherence.

## Introduction

Non-adherence to treatments for cardiovascular disease (CVD) is an increasingly-recognized cause of adverse CVD outcomes in adults with CVD [1, 2]. For example, non-adherence to anti-hypertensives and statins in patients with chronic coronary artery disease was associated with 10–40% increased risk of cardiovascular hospitalizations and 50–80% increased risk of death [3], while non-adherence to statins in patients within a year after hospitalization for myocardial infarction was associated with 12–25% increased risk of death [4].

Breast cancer patients are a unique population facing higher risk of CVD while navigating through their breast cancer diagnosis and treatment. Thus, managing chronic and preventive medications during and after treatment for breast cancer has become increasingly important. Prior studies in breast cancer survivors have shown that adherence to commonly prescribed CVD medications is poor, particularly for statins and diuretics during breast cancer treatment and in the year following diagnosis [5, 6]. This behavior is likely driven by the stronger emphasis on effectively treating the breast cancer by both clinicians and patients.

Statins are the most commonly used lipid-lowering medication, and well-established for preventing CVD in the general population [7, 8]. However, evidence on the effect of adherence to statins on risk of CVD in breast cancer survivors is limited, especially in the context of survivors concurrently progressing through breast cancer diagnosis and treatment. Furthermore, studies to date lack a methodological approach with proper consideration, in a causal inference framework, of time-dependent confounding of clinical risk factors on risk of CVD outcomes. At Kaiser Permanente Northern California (KPNC), an integrated healthcare system, complete capture of pharmacy data exists within the electronic health record (EHR), along with capture of CVD risk factors and outcomes and breast cancer clinical characteristics and treatment. Recognizing these strengths, we primarily assessed adherence to statins, along with exploring anti-hypertensive and diabetes medications, and their association with major CVD events over 14 years of follow-up in breast cancer patients with dyslipidemia, hypertension, and diabetes, respectively, at KPNC.

## Materials and methods

### Overview

The Pathways Heart Study is an ongoing cohort study in KPNC examining the incidence of CVD events and cardiometabolic risk factors in women with and without a history of breast cancer. KPNC is a comprehensive healthcare system providing clinical care to over 4.5 million members in the San Francisco Bay, Sacramento, and Central Valley metropolitan areas. Data were initially accessed for research purposes on June 11, 2019. Authors at KPNC had access to information that could identify individual participants during or after data collection.

This study was performed in line with the principles of the Declaration of Helsinki. Approval was granted by the Human Subjects Institutional Review Board (IRB) of Kaiser Permanente Northern California (current approval until June 11, 2025). This is a data-only study which used existing protected health information in the KPNC electronic health record. Therefore, waiver of informed consent was not required as data analysis is regulated by the HIPAA Privacy Rule.

### Study population

**Fig 1:**
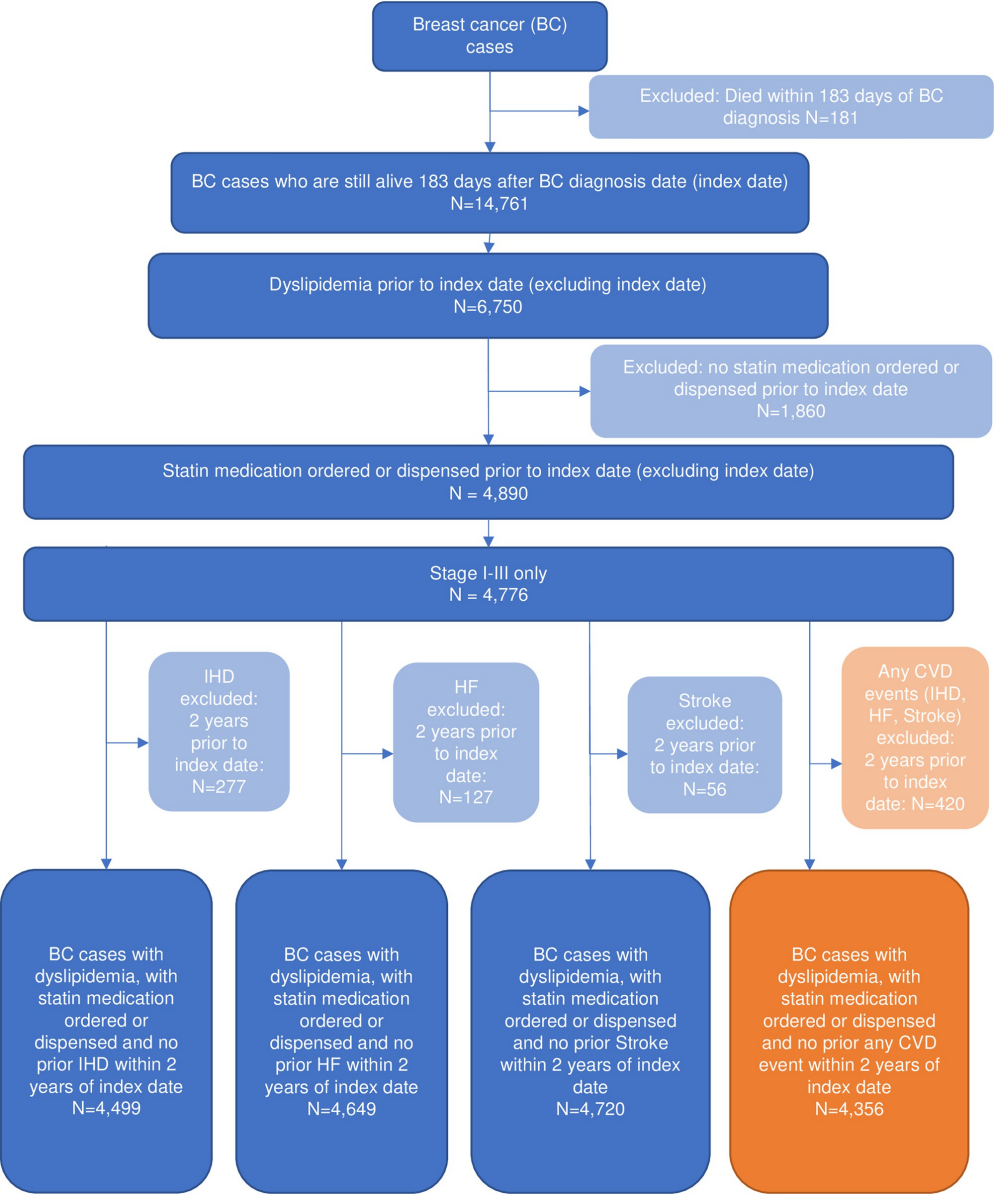
Consort diagram for dyslipidemia cohort, Pathways Heart Study.

Female patients with breast cancer had a new diagnosis of stage I-III breast cancer between 2005–2013 at KPNC and had no previous history of invasive cancer. They were 21 years or older and had continuous KPNC membership ≥12 months prior to and on the diagnosis date, allowing for up to a 31-day gap in membership. To be included in this analysis, they had to be alive on the 183^rd^ day following breast cancer diagnosis (index date of cohort entry after cohort average of chemotherapy completion) and have a prior diagnosis of dyslipidemia (or hypertension or diabetes) in the 30 months before the index date, along with ≥1 prior outpatient pharmacy order or dispensing for an indicated medication in the 30 months before the index date. Indicated medications included statins for dyslipidemia, anti-hypertensive medications with diuretics for hypertension, and oral hypoglycemic agents and insulin to control Type 1 and 2 diabetes. Follow-up started at day 183 after breast cancer diagnosis and ended at the earliest of study outcome occurrence, or one of the following three right-censoring events: disenrollment from the KP health plan, death, or administrative end of study (12/31/2018). Consolidated Standards of Reporting Trials (CONSORT) diagrams are available for the identified cohorts of breast cancer women with history of dyslipidemia (n = 4,776, Fig 1), hypertension (n = 6,240, S1 Fig), and diabetes (n = 1,738, S2 Fig).

Administrative and clinical data were extracted from KPNC electronic health records (EHR), with information for more than 4.5 million members across 21 hospitals and 262 outpatient clinics in the Northern California region. Tumor and treatment characteristics were obtained from the KPNC Cancer Registry that reports to the NCI’s Surveillance, Epidemiology, and End Results (SEER) program.

### CVD medication exposure

Exposure to statins, anti-hypertensives, and diabetes medications were obtained from medication dispensing data in the KPNC outpatient pharmacy database. These data included fill dates, drug type, and days’ supply. A comprehensive list of generic drug names is available upon request.

For each patient, adherence to medications was represented by a time-varying exposure profile by mapping each dispensing into a drug prescription coverage period during which the patient is determined to be at least 80% adherent. To determine 80% adherent drug coverage periods, the episode start date was the fill date and the episode end date was the fill date plus day supply multiplied by 1.25 (inverse of 80%). No stockpiling and no gap bridging were assumed. Specifically, no stockpiling was tracked when a new prescription was filled before the end date of the prior episode and all drugs from the prior episode were assumed to be used, and no gap bridging between prescription coverage periods was applied when a new prescription was filled after the end date of the prior prescription.

### Covariates

Covariate data were extracted from the EHR and included breast cancer clinical and treatment characteristics, laboratory values, health status and behaviors, sociodemographic characteristics, and CVD risk factors and conditions at cohort entry (S1 Table). All these covariates were considered potential confounders and included in the statistical analysis as described below.

Specifically, clinical and treatment characteristics of the original breast tumor included American Joint Committee on Cancer (AJCC) stage, estrogen receptor, progesterone receptor, and HER2 status, type of breast surgery, and receipt of chemotherapy, radiation therapy, or endocrine therapy. Laboratory values collected were Hemoglobin A1C (HbA1c), high-density lipoprotein (HDL), low-density lipoprotein (LDL), total cholesterol, creatinine, and triglycerides. Health status and behaviors included diastolic and systolic blood pressure values, menopausal status, and body mass index (BMI). The Comorbidity Point Score Version 2 (COPS2) [9] is a measure of comorbidity calculated using diagnosis codes of inpatient and outpatient encounters that occurred in the year prior to diagnosis or reference date. Smoking history included never, former, and current smoker, and census-based socioeconomic (SES) measures included percent households below poverty, geocoded household income, and low education (up to 12th grade but not high school graduate).

### Cardiometabolic risk factors

The three cardiometabolic risk factors included dyslipidemia, hypertension, and diabetes. Prior history of dyslipidemia at baseline was defined as: 1) 2 separate diagnosis codes of International Classification of Diseases, Ninth Revision (ICD-9-CM) 272.0–272.4 or Tenth Revision (ICD-10-CM) E78.00, E78.01, E78.1-R78.5; 2) diagnosis code and abnormal lab test results (LDL cholesterol ≥160 mg/dL); 3) diagnosis code and dispensed lipid-lowering medication such as statins and other antilipemic agents; or 4) dispensed lipid-lowering medication and abnormal lab test results (LDL cholesterol ≥160 mg/dL).

Prior history of hypertension at baseline was defined according to the KPNC Preventing Heart Attacks and Strokes Everyday (PHASE) program [10]. Criteria were: 1) 2 or more essential (primary) hypertension diagnoses of ICD-9-CM 401.xx or ICD-10-CM I10, I16.0, I16.1, I16.9) during primary care visits in the prior 2 years; 2) 1 or more primary care hypertension diagnoses and 1 or more hospitalizations with a primary or secondary hypertension diagnosis in the prior 2 years; or 3) 1 or more primary care hypertension diagnoses and 1 or more filled prescriptions for hypertension medication within the prior 6 months.

Prior history of diabetes at baseline was identified from the KPNC Diabetes Registry [11]. Inclusion criteria are any one of the following: 1) at least 1 principal inpatient diagnosis of ICD-9-CM 250.xx or ICD-10-CM E10.xxx, E11.xxx, E13.xxx, O24.xxx; 2) at least 2 outpatient diagnoses spanning the current and previous five years; 3) 2 or more abnormal lab test results on separate days spanning the current year and the last two years (i.e., fasting blood glucose ≥ 126 mg/dL); 4) at least 1 diabetes medication prescription such as insulin, oral hypoglycemics or insulin sensitizers. Patients with only one of the indicators must have additional indicators during the subsequent two years of KPNC membership.

### CVD outcomes

The four study outcomes included heart failure (HF), ischemic heart disease (IHD), stroke, and a composite CVD outcome of HF, IHD and stroke. They were ascertained according to ICD-9-CM and ICD-10-CM diagnosis codes and Current Procedural Terminology (CPT4®) Codes. A physician-adjudicated validation study on the ICD diagnosis codes was completed in 337 breast cancer patients and found positive predictive values of code ascertainment versus chart review validation ranging from 89 to 94% [12]. The complete list of codes is in S2 Table.

Death data through 2018 were obtained from linkage to the KPNC mortality file, which is regularly updated with data from the California State Department of Vital Statistics, U.S. Social Security Administration, National Death Index, and KPNC membership and utilization databases.

### Statistical analysis

Longitudinal analytic datasets were assembled for each cohort and CVD outcome using the %_MSMstructure SAS macro [13] to track the temporal ordering of covariate, exposure, outcome, and right-censoring event measurements, updated every 90 days between the index date and end of follow-up. Using these datasets, the effect of medication adherence on the risk of the outcomes was evaluated using working marginal structural models (MSM) [14, 15] for the discrete-time hazard function fitted with inverse probability weighting to address both baseline and time-varying sources of confounding and selection bias (attrition bias) [16]. Three working logistic MSMs for the discrete-time hazards were fitted to each outcome and cohort separately to calculate hazard ratios (HR) and 95% confidence intervals (CI). Each parameterization included separate terms for each quarter of follow-up and one or two exposure terms for the cumulative average adherence (CAA) experienced by the patient between baseline and each quarter of follow-up. The three MSMs differed with respect to categorizing the continuous cumulative average adherence: 1) CAA<100% versus CAA = 100% (reference), 2) CAA<80% versus CAA≥80% (reference) and 3) CAA<80% versus 80%≤CAA<100% versus CAA = 100%.

The propensity scores for exposure and censoring to define the inverse probability weights used to fit each MSM were estimated using a data-adaptive estimation called Super Learning [17]. Super Learning is an ensemble learning method used for both adapting the covariate adjustment set that best estimates the propensity scores (see S1 Table for covariate selection) and allowing for complex, nonlinear associations between covariates and exposure/censoring. Thus, Super Learning consists of combining estimated values from various candidate learners through a weighted average. The selection of the optimal combination of learners is based on cross-validation to protect against overfitting such that the resulting learner (called super learner) performs asymptotically as well as or better than any of the candidate learners considered. We considered the following learners: 5 main-term-only logistic models (glm) that each includes the last measurement of all covariates or only the first 5, 10, 20, and 30 covariates most associated with the exposure or censoring event, 2 linear splines with tensor products (polyclass) [18], 2 random forests (rngr), and 2 extreme gradient boosting regressions (xgb) [19, 20]. Each of the two polyclass, rng and xgb learners considered only the first 5 or 10 covariates most associated with the exposure or censoring event. Inverse probability weights were stabilized and truncated using two cutoff levels to evaluate the sensitivity of findings to the truncation level chosen (either the value 20 or the 99^th^ percentile of the inverse probability weight values) [21, 22]. Standard error estimates were derived using the influence curves of the inverse probability weighting estimators for the coefficients of the logistic working MSMs [23]. We used the missingness indicator approach to handle partially missing baseline and time-dependent covariate values [24–27].

## Results

In the dyslipidemia cohort, women were on average 67 years old at breast cancer diagnosis with BMI of 30 kg/m^2^ and 65% non-Hispanic white (Table 1). Half were non-smokers (50.2%) and most were post-menopausal (94.5%). They had a history of CVD risk factors including hypertension (72.2%) and diabetes (33.8%). A small proportion (8.8%) experienced a major CVD event before their breast cancer diagnosis.

**Table 1:** Characteristics of early-stage (AJCC I-III) breast cancer (BC) patients diagnosed with dyslipidemia and prescribed a statin (dyslipidemia cohort), Pathways Heart Study.

	Dyslipidemia Cohort	
	(n = 4,776)	
	mean	sd
**Length of KPNC membership (months)**	205	57
**COPS2 comorbidity score at BC diagnosis**	18	19
**BMI (kg/m** ^**2**^ **) at BC diagnosis**	30	6
**Age (years) at BC diagnosis**	67	10
**Median neighborhood household income ($)**	78,644	34,071
** **	**n**	**%**
**Race/ethnicity**		
White	3,104	64.99
Black	379	7.94
Asian	681	14.26
Hispanic	556	11.64
Pacific Islander	24	0.50
American Indian / Alaskan Native	32	0.67
**Smoking status at BC diagnosis**		
Never smoker	2,398	50.21
Quit/former smoker	1,492	31.24
Current smoker	463	9.69
Unknown	423	8.86
**Menopausal Status at BC diagnosis**		
Post-menopausal	4,512	94.47
Pre-menopausal	264	5.53
**AJCC Stage**		
I	2,835	59.36
II	1,515	31.72
III	426	8.92
**Received Chemotherapy**	1,747	36.58
**Received Radiation Therapy**	3,038	63.61
**Received Endocrine Therapy**	3,551	74.35
**History of dyslipidemia before BC**	--	--
**History of hypertension before BC**	3,450	72.24
**History of diabetes before BC**	1,615	33.81
**History of ischemic heart disease before BC**	277	5.80
**History of heart failure before BC**	127	2.66
**History of stroke before BC**	56	1.17
**History of any CVD before BC**	420	8.79

**Fig 2:**
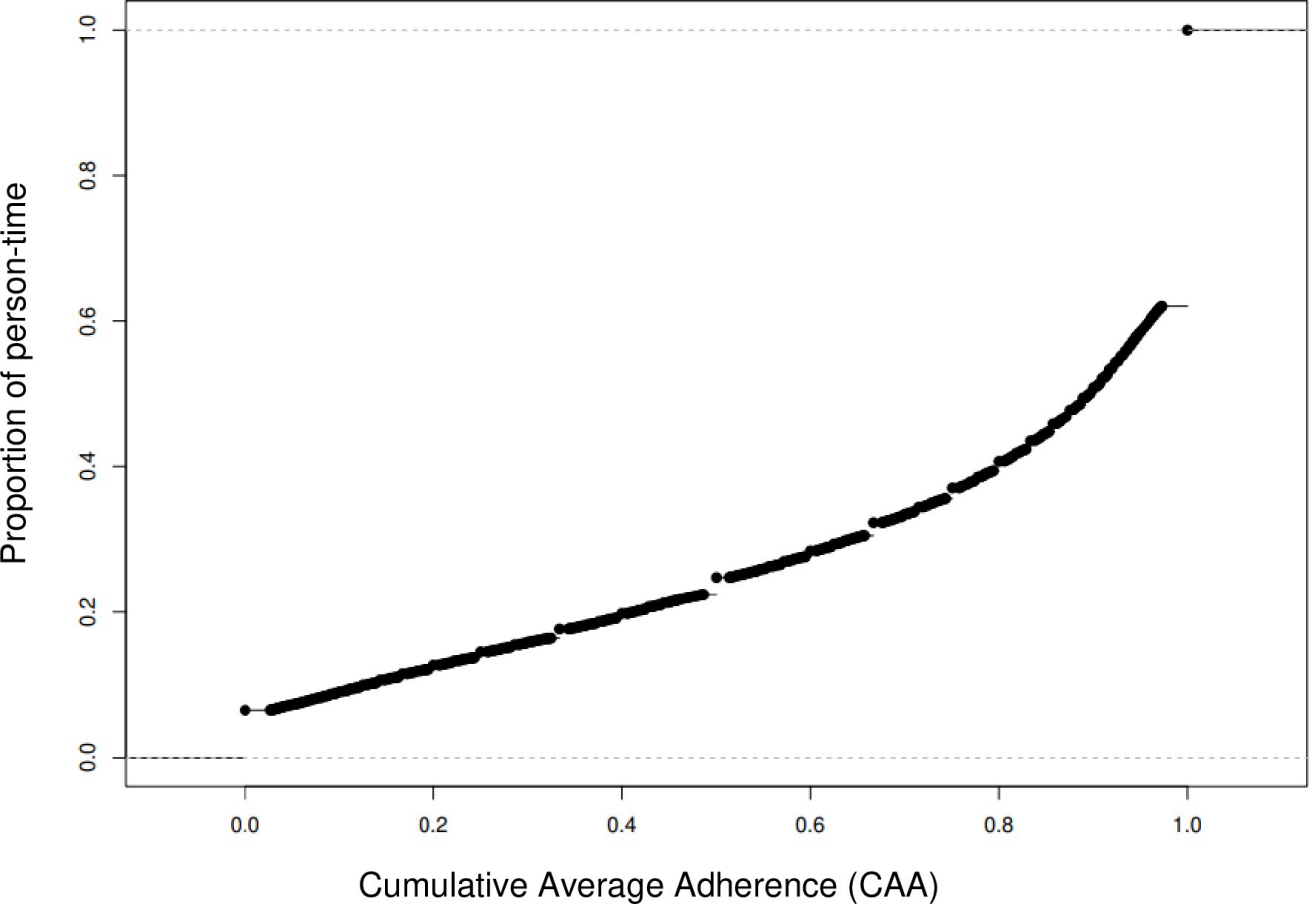
Distribution of cumulative average adherence (CAA) of statin use in the dyslipidemia cohort (n = 4,776), pathways study.

During a mean follow-up of 6.5 years 420 HF, 360 IHD, and 170 stroke events occurred, and 698 women experienced at least one of the three CVD events in the dyslipidemia cohort (Table 2). The distribution of the CAA in the cohort over study follow-up is shown in Fig 2, with about 63% of the cohort having 100% perfect adherence. In general, poor statin adherence (CAA<80%) was significantly associated with 2.1–2.5 times higher risk of composite CVD for most adherence categorizations: CAA<80% versus CAA≥80% (HR = 2.54; 95% CI: 1.09–5.94) and CAA<100% versus CAA = 100% (HR = 2.08; 95% CI: 0.93–4.54). For dose response of adherence, compared with perfect adherence (CAA≥100%), CAA<80% had similar higher risk (HR = 2.45; 95% CI: 1.05–5.70) of composite CVD, yet good adherence (80%≤CAA<100%) had lower risk (HR = 0.35; 95% CI: 0.13–0.92). For the individual CVD outcomes, only stroke, but not IHD or HF, was associated with adherence level. Specifically, CAA<80% versus CAA≥80% was associated with higher risk of stroke (HR = 8.13; 95% CI: 2.03–32.51) as well as CAA<100% versus CAA = 100% (HR = 7.06; 95% CI: 1.78, 27.97). For dose response, compared with perfect adherence (CAA = 100%), CAA<80% had similar higher risk of stroke (HR = 8.00; 95% CI: 2.00–32.05), yet good adherence (80%≤CAA<100%) had lower, yet non-significant, risk (HR = 0.55; 95% CI: 0.18–1.69).

**Table 2:** Adherence to statins and risk of CVD event in the Pathways Heart Study.

Outcome	MSM Parameter-ization	Person-Time	Reference Exposure*	%†	Non-reference Exposure*	%†	Super learner HR (95% CI)‡
**Heart Failure (n = 420)**	1	122,793	CAA = 100%	63	CAA<100%	37	0.90 (0.27, 3.00)
	2	122,793	CAA≥80%	39	CAA<80%	61	1.03 (0.31, 3.48)
	3	122,793	CAA = 100%	39	CAA<80%	23	1.00 (0.30, 3.42)
					0.8≤CAA<100%	37	**0.32 (0.11, 0.92)**
**Ischemic Heart Disease (n = 360)**	1	117,491	CAA = 100%	63	CAA<100%	37	2.13 (0.75, 5.99)
	2	117,491	CAA≥80%	40	CAA<80%	60	2.69 (0.85, 8.52)
	3	117,491	CAA = 100%	40	CAA<80%	23	2.57 (0.83, 7.99)
					80%≤CAA<100%	37	0.37 (0.10, 1.37)
**Stroke (n = 170)**	1	126,554	CAA = 100%	63	CAA<100%	37	**7.06 (1.78, 27.97)**
	2	126,554	CAA≥80%	39	CAA<80%	61	**8.13 (2.03, 32.51)**
	3	126,554	CAA = 100%	39	CAA<80%	24	**8.00 (2.0, 32.05)**
					80%≤CAA<100%	37	0.55 (0.18, 1.69)
**All CVD (n = 698) **	1	110,396	CAA = 100%	62	CAA<100%	38	2.08 (0.93, 4.64)
	2	110,396	CAA≥80%	39	CAA<80%	61	**2.54 (1.09, 5.94)**
	3	110,396	CAA = 100%	39	CAA<80%	23	**2.45 (1.05, 5.70)**
					80%≤CAA<100%	38	**0.35 (0.13, 0.92)**

* Exposure level indicates a patient’s cumulative average adherence (CAA) up to the risk set that defines each hazard ratio (HR) and 95% confidence interval (CI)

† Proportion of cohort in exposed group

‡ Stabilized, truncated at 20, inverse probability weighting HR estimates. An HR>1 indicates that the non-reference exposure level is deleterious.

In other analyses, for the hypertension cohort, over a mean follow-up of 6.5 years 554 HF, 438 IHD, 248 stroke events occurred, and 921 women experienced any CVD event (Table 3). Adherence to anti-hypertensives was generally not significantly associated with risk of HF, IHD, or composite CVD. For the diabetes cohort, during a mean follow-up of 6.5 years 93 HF, 149 IHD, 66 stroke events occurred, and 291 women experience any CVD event (Table 4). Adherence to diabetes medications was generally not significantly associated with risk of HF, stroke, or composite CVD as well.

**Table 3:** Adherence to anti-hypertensive medications and risk of CVD event in the Pathways Heart Study.

Outcome	MSM Parameter-ization	Person-Time	Reference Exposure*	%†	Non-reference Exposure*	%†	Super learner HR (95% CI)‡
**Heart Failure (n = 554)**	1	158,242	CAA = 100%	47	CAA<100%	53	1.47 (0.57–3.77)
	2	158,242	CAA≥80%	24	CAA<80%	76	1.46 (0.51–4.14)
	3	158,242	CAA = 100%	24	CAA<80%	23	1.48 (0.52–4.21)
					0.8≤CAA<100%	53	1.42 (0.33–6.13)
**Ischemic Heart Disease (n = 438)**	1	154,243	CAA = 100%	47	CAA<100%	53	1.70 (0.73–3.95)
	2	154,243	CAA≥80%	24	CAA<80%	76	1.42 (0.52–3.83)
	3	154,243	CAA = 100%	24	CAA<80%	23	1.51 (0.56–4.12)
					80%≤CAA<100%	53	2.43 (0.68–8.66)
**Stroke (n = 248)**	1	163,222	CAA = 100%	47	CAA<100%	53	**0.30 (0.11–0.83)**
	2	163,222	CAA≥80%	24	CAA<80%	76	0.36 (0.12–1.12)
	3	163,222	CAA = 100%	24	CAA<80%	23	0.35 (0.11–1.08)
					80%≤CAA<100%	53	**0.14 (0.03–0.57)**
**All CVD (n = 921)**	1	144,569	CAA = 100%	46	CAA<100%	54	1.21 (0.58–2.54)
	2	144,569	CAA≥80%	24	CAA<80%	76	0.91 (0.37–2.23)
	3	144,569	CAA = 100%	24	CAA<80%	22	0.96 (0.39–2.35)
					80%≤CAA<100%	54	2.50 (0.85–7.40)

* Exposure level indicates a patient’s cumulative average adherence (CAA) up to the risk set that defines each hazard ratio (HR) and 95% confidence interval (CI)

† Proportion of cohort in exposed group

‡ Stabilized, truncated at 20, inverse probability weighting HR estimates. An HR>1 indicates that the non-reference exposure level is deleterious.

**Table 4:** Adherence to diabetes medications (oral hypoglycemic agents or insulin) and risk of CVD event in the Pathways Heart Study.

Outcome	MSM Parameter-ization	Person-Time	Reference Exposure*	%†	Non-reference Exposure*	%†	Super learner HR (95% CI)‡
**Heart Failure (n = 193)**	1	40,736	CAA = 100%	65	CAA<100%	35	0.83 (0.22–3.15)
	2	40,736	CAA≥80%	40	CAA<80%	60	0.96 (0.27–3.50)
	3	40,736	CAA = 100%	40	CAA<80%	25	0.91 (0.24–3.43)
					0.8≤CAA<100%	35	0.27 (0.05–1.37)
**Ischemic Heart Disease (n = 149)**	1	39,317	CAA = 100%	65	CAA<100%	35	**0.15 (0.05–0.47)**
	2	39,317	CAA≥80%	40	CAA<80%	60	**0.12 (0.04–0.42)**
	3	39,317	CAA = 100%	40	CAA<80%	25	**0.12 (0.03–0.41)**
					80%≤CAA<100%	35	0.33 (0.09–1.23)
**Stroke (n = 66)**	1	43,108	CAA = 100%	66	CAA<100%	34	5.23 (0.87–31.60)
	2	43,108	CAA≥80%	41	CAA<80%	59	6.06 (0.90–40.74)
	3	43,108	CAA = 100%	41	CAA<80%	25	6.15 (0.90–42.13)
					80%≤CAA<100%	34	1.16 (0.21–6.36)
**All CVD (n = 291) **	1	36,429	CAA = 100%	64	CAA<100%	36	0.43 (0.15–1.26)
	2	36,429	CAA≥80%	39	CAA<80%	61	0.52 (0.17–1.59)
	3	36,429	CAA = 100%	39	CAA<80%	25	0.48 (0.16–1.49)
					80%≤CAA<100%	36	**0.21 (0.06–0.70)**

* Exposure level indicates a patient’s cumulative average adherence (CAA) up to the risk set that defines each hazard ratio (HR) and 95% confidence interval (CI)

† Proportion of cohort in exposed group

‡ Stabilized, truncated at 20, inverse probability weighting HR estimates. An HR>1 indicates that the non-reference exposure level is deleterious.

## Discussion

In this prospective study of breast cancer survivors, maintaining good adherence of at least 80% to statins after breast cancer treatment is overall beneficial for cardiovascular health in patients with dyslipidemia. Poor cumulative average adherence of less than 80% was associated with 2.5 times higher risk of composite CVD which was driven separately by 2.7 times higher risk of IHD and 8.1 times higher risk of stroke. In contrast, risk of HF was not affected by lower adherence to statins after breast cancer treatment. Results for adherence to anti-hypertensives and diabetes medications were mixed showing no definitive associations for risk of CVD outcomes.

Our findings are generally consistent with the established evidence that CVD medications are the most common medical intervention for prevention of CVD through modification of intermediate determinants of CVD including lipid control [28, 29], blood pressure control [29, 30], and glycemic control [31, 32], yet patient adherence to statins, anti-hypertensives, and diabetes medications is equally important. Given our observed negative impact of non-adherence on CVD health in women with breast cancer, efforts to improve CVD medication adherence in this population are important to pursue. A recent systematic review of adherence to CVD medications in patients with cancer underscored that medication nonadherence is a prevalent and multifactorial problem [33]. The authors suggested informing survivors of the importance of taking their medications as prescribed and to provide resources, as appropriate, to support survivors in achieving this goal. In addition, incorporating assessment of adherence into the care management of survivors might be beneficial. In fact, a recent study examined the effects of changes in the provider team structure on changes in adherence during and after cancer treatment, and found that provider team structure only explained a small portion of changes in medication adherence [34]. Thus, future studies identifying methods to improve CVD medication adherence might need to focus on larger systemic and patient factors across primary care, oncology, and cardiology.

Prior studies on adherence to CVD medications in women diagnosed with early-stage breast cancer who were users at time of diagnosis have generally reported decreases in adherence from pre- to post-cancer diagnosis [33]. Two studies [35, 36] reported adherence rates at two years post-diagnosis of 39% and 71% for statins or lipid-lowering medications, 37% and 86% for anti-hypertensives, and 74% and 75% for diabetes medications. Several prior studies have been conducted in integrated health care settings such as ours with access to outpatient pharmacy data. For statins, one study at KP Northwest found no reduction in adherence to statins over two years post-diagnosis (~67%) as measured by proportion of days covered (PDC) [37]. In contrast, another study at KP Washington reported reductions in adherence as measured by medication possession ratio (MPR) for both statins and diabetes medications over three years post- breast cancer treatment, specifically lowest proportions of 35.9% for statins and 24.6% for diabetes medications at post-two years [5, 38]. Another study of 36,149 early-stage breast cancer survivors using MarketScan data from 2009–2013 also found decreased adherence as measured by MPR to lipid-lowering medications, including statins, in the first year after breast cancer treatment [6]. To our knowledge, only one study to date, and also from our group, used SEER-Medicare data to examine the impact of adherence to lipid-lowering medications after breast cancer diagnosis and risk of CVD [39]. We found that 15,576 early-stage breast cancer patients who were prevalent CVD medication users at diagnosis had a 21% increased risk of experiencing a cardiac event (acute ischemic event or acute HF) with non-adherence (MPR<80%) to lipid-lowering medications [39].

Most prior adherence studies of CVD medications, including ours from 2020 [39], aggregated medication exposures over an entire study period using MPR and PDC derived from pharmacy data [33]. While both MPR and PDC are standard measures reporting percentage of time when a patient has a medication available, a substantial limitation is the inability to address time-dependent confounding. Herein, we overcame this limitation by conducting analyses to evaluate the effects of sequences of adherence measures updated every 90 days (adherent at the 80% level yes/no). We then used inverse probability weighting to fit working MSMs and evaluate the effect of these sequences relating outcome risk to the average of the past medication adherence sequence. Our approach combined a rigorous causal inference framework with machine learning for flexible adjustment for confounding and sources of selection bias.

One study limitation is while we attempted to account for all underlying confounders in the association between adherence to statins, anti-hypertensives, and diabetes medications and CVD outcomes, unmeasured confounding cannot be ruled out. For example, we did not account for duration of each cardiometabolic risk factor (dyslipidemia, hypertension, diabetes) experienced by the patient. We also did not consider clinician characteristics and communication style, social determinants of health of the patient, and physiological responses of the patient to breast cancer and its treatment. This constraint might help explain why we found that good adherence to statins (≥80%), rather than perfect adherence (100%), was associated with the lowest risk of any CVD in the dose response models. The fitted MSMs also produced unstable effect estimates with wide confidence intervals, most likely due to limited CVD events in the hypertension and diabetes cohorts.

## Conclusion

Overall, maintaining good adherence (≥80%) to statins was protective against CVD, specifically stroke, yet unmeasured confounding remains a concern. Findings from this study on variable medication adherence to CVD medications over the survivorship course further emphasize the necessity of quality survivorship care in breast cancer patients.

## Supporting information

S1 Fig Consort diagram for hypertension cohort, Pathways Heart Study. (DOCX)

S2 Fig Consort diagram for diabetes cohort, Pathways Heart Study. (DOCX)

S1 Table List of covariates included in the working marginal structural models. (DOCX)

S2 Table Standard codes for the ascertainment of non-fatal and fatal major cardiovascular events in the Pathways Heart Study. (DOCX)

## Abbreviations

AJCC: American Joint Committee on Cancer
BC: breast cancer
BMI: body mass index
CAA: cumulative average adherence
CI: confidence interval
CONSORT: Consolidated Standards of Reporting Trials
COPS2: Comorbidity Point Score Version 2
CVD: cardiovascular disease
EHR: electronic health record
HF: heart failure
HR: hazard ratio
IHD: ischemic heart disease
KPNC: Kaiser Permanente Northern California
MPR: medication possession ratio
MSM: marginal structural model
PDC: proportion of days covered
PHASE: Preventing Heart Attacks and Strokes Everyday
SEER: Surveillance, Epidemiology, and End Results
SES: socioeconomic status
