# Supplementary material for: Adherence to cardiovascular medications and risk of cardiovascular disease in breast cancer patients: A causal inference approach in the Pathways Heart Study
Source: PLoS One. 2024 Sep 19;19(9):e0310531. doi: 10.1371/journal.pone.0310531 (PMC11412667; doi:10.1371/journal.pone.0310531)
Supplement: S1 Fig — (DOCX) [file pone.0310531.s001.docx]

# **Supplemental Figure 1. Consort Diagram for Hypertension Cohort, Pathways Heart Study**

BC cases who are still alive 183 days after BC diagnosis date (index date)

N=14,761

Hypertension from PHASE prior to index date (excluding index date)

N=7,093

Antihypertensive medication ordered or dispensed prior to index date (excluding index date)

N = 6,438

BC cases with Hypertension, with antihypertensive medication ordered or dispensed and No prior IHD within 2 years of index date

N=5,961

BC cases with Hypertension, with antihypertensive medication ordered or dispensed and No prior HF within 2 years of index date

N=6,061

BC cases with Hypertension, with antihypertensive medication ordered or dispensed and No prior Stroke within 2 years of index date

N=6,179

Excluded: Died within 183 days of BC diagnosis N=181

IHD excluded:

2 years prior to index date: N=279

HF excluded:

2 years prior to index date:

N=179

Stroke excluded:

2 years prior to index date:

N=61

BC cases with Hypertension, with antihypertensive medication ordered or dispensed and No prior Any CVD event within 2 years of index date

N=5,770

Any CVD events (IHD, HF, Stroke) excluded:

2 years prior to index date: N=470

Breast cancer (BC) cases
N=14,942

Excluded: no antihypertensive medication ordered or dispensed prior to index date

N=655

Stage I-III only

N = 6,240
