# Supplementary material for: Adherence to cardiovascular medications and risk of cardiovascular disease in breast cancer patients: A causal inference approach in the Pathways Heart Study
Source: PLoS One. 2024 Sep 19;19(9):e0310531. doi: 10.1371/journal.pone.0310531 (PMC11412667; doi:10.1371/journal.pone.0310531)
Supplement: S2 Fig — (DOCX) [file pone.0310531.s002.docx]

# **S2 Fig. Consort Diagram for Diabetes Cohort, Pathways Heart Study**

BC cases who are still alive 183 days after BC diagnosis date

N=14,761

Diabetes diagnosed prior to index date (excluding index date)

N=2,130

Insulin or OHA medication ordered or dispensed prior to index date (excluding index date)

N = 1,793

BC cases with Diabetes, with OHA or Insulin medication ordered or dispensed and No prior IHD **within** 2 years of index date

N=1,630

BC cases with Diabetes, with OHA or Insulin medication ordered or dispensed and No prior HF **within** 2 years of index date

N=1,661

BC cases with Diabetes, with OHA or Insulin medication ordered or dispensed and No prior Stroke within 2 years of index date

N=1,716

Excluded: Died within 183 days of BC diagnosis N=181

IHD excluded:

2 years prior to index date: N=108

HF excluded:

2 years prior to index date:

N=77

Stroke excluded:

2 years prior to index date:

N=22

BC cases with Diabetes, with OHA or Insulin medication ordered or dispensed and No prior Any CVD event within 2 years of index date

N=1,553

Any CVD events (IHD, HF, Stroke) excluded:

2 years prior to index date: N=185

Breast cancer (BC) cases
N=14,942

Excluded: no Insulin or OHA medication ordered or dispensed prior to index date

N=337

Stage I-III

N = 1,738
