# Supplementary material for: Adherence to cardiovascular medications and risk of cardiovascular disease in breast cancer patients: A causal inference approach in the Pathways Heart Study
Source: PLoS One. 2024 Sep 19;19(9):e0310531. doi: 10.1371/journal.pone.0310531 (PMC11412667; doi:10.1371/journal.pone.0310531)
Supplement: S1 Table — (DOCX) [file pone.0310531.s003.docx]

**S1Table.** **List of covariates included in the working marginal structural models**

|  | **Time-varying or Fixed** | **Continuous or Categorical** |
| --- | --- | --- |
| **Clinical and Treatment Characteristics of Breast Cancer** |  |  |
| Age at breast cancer diagnosis | Fixed | Continuous |
| AJCC stage | Fixed | Categorical |
| Metastases | Fixed | Categorical |
| Estrogen receptor status | Fixed | Categorical |
| Progsterone receptor status | Fixed | Categorical |
| Estrogen and progesterone statuses combined | Fixed | Categorical |
| HER2 status | Fixed | Categorical |
| Triple negative status | Fixed | Categorical |
| Laterality | Fixed | Categorical |
| Surgery received (Y/N) | Time-varying | Categorical |
| Chemotherapy received (Y/N) | Time-varying | Categorical |
| Radiation therapy received (Y/N) | Time-varying | Categorical |
| Endocrine therapy received (Y/N) | Time-varying | Categorical |
|  |  |  |
| **Laboratory Values (at baseline)** |  |  |
| HGBA1C | Time-varying | Continuous |
| Cholesterol | Time-varying | Continuous |
| HDL | Time-varying | Continuous |
| LDL | Time-varying | Continuous |
| Creatinine | Time-varying | Continuous |
| Triglycerides | Time-varying | Continuous |
|  |  |  |
| **Health Status and Behaviors** |  |  |
| Menopausal status | Fixed | Categorical |
| Body mass index (BMI) | Time-varying | Categorical |
| Comoribidty Point Score Version 2 (COPS2) | Time-varying | Continuous |
| Smoking history | Fixed | Categorical |
|  |  |  |
| **Sociodemographic Characteristics** |  |  |
| Race/ethnicity | Fixed | Categorical |
| Percent households below poverty level income | Fixed | Continuous |
| Geocoded household income | Fixed | Continuous |
| Low education (Y/N) | Fixed | Categorical |
|  |  |  |
| **CVD Risk Factors and Conditions** |  |  |
| Dyslipidemia (Y/N) | Time-varying | Categorical |
| Hypertension (from blood pressure) (Y/N) | Time-varying | Categorical |
| Hypertension (PHASE definition) (Y/N) | Time-varying | Categorical |
| Diabetes (Y/N) | Time-varying | Categorical |
| Heart Failure prior to breast cancer (Y/N) | Time-varying | Categorical |
| Ischemic Heart Disease prior to breast cancer (Y/N) | Time-varying | Categorical |
| Stroke prior to breast cancer (Y/N) | Time-varying | Categorical |
|  |  |  |
| **CVD Medications** |  |  |
| Dyslipidemia treatment (any) (Y/N) | Time-varying | Categorical |
| Dyslipidemia treatment (non-statin) (Y/N) | Time-varying | Categorical |
| Hypertension treatment (any) (Y/N) | Time-varying | Categorical |
| Diabetes treatment (any) (Y/N) | Time-varying | Categorical |
| Diabetes treatment (non-insulin) (Y/N) | Time-varying | Categorical |
| Diabetes treatment (non-OHA) (Y/N) | Time-varying | Categorical |
| Diabetes treatment (non-insulin & non-OHA) (Y/N) | Time-varying | Categorical |
