# Supplementary material for: Adherence to cardiovascular medications and risk of cardiovascular disease in breast cancer patients: A causal inference approach in the Pathways Heart Study
Source: PLoS One. 2024 Sep 19;19(9):e0310531. doi: 10.1371/journal.pone.0310531 (PMC11412667; doi:10.1371/journal.pone.0310531)
Supplement: S2 Table — (DOCX) [file pone.0310531.s004.docx]

**S2 Table.** **Standard codes for the ascertainment of non-fatal and fatal major cardiovascular events in the Pathways Heart Study**

|  | **Primary International Classification of Diseases, Ninth Revision, Clinical Modification (ICD-9-CM) Diagnosis or Procedure Codes** | **Primary International Classification of Diseases, Tenth Revision, Clinical Modification (ICD-10-CM) Diagnosis or Procedure Codes** | **Current Procedural Terminology (CPT4®) Codes for Medical Billing Services** |
| --- | --- | --- | --- |
| **Ischemic Heart Disease** |  |  |  |
| 1. Acute myocardial infarction | 410.X | I21.01, I21.02, I21.3, I21.4, I21.09, I21.11, I21.19, I21.21, I21.29, I22.0, I22.1, I22.2, I22.8, I22.9 | Not applicable |
| 1. Other acute & subacute forms of ischemic heart disease | 411.X | I20.0, I24.0, I24.1, I24.8, I24.9, I25.720, I25.750, I25.760, I25.790 | Not applicable |
| 1. Angina pectoris | 413.X | I20.1, I20.8, I20.9, I25.110,I25.111, I25.118, I25.119, I25.701, I25.708, I25.709, I25.711, I25.718, I25.719, I25.721, I25.728, I25.729, I25.730, I25.731, I25.738, I25.739, I25.751, I25.758, I25.759, I25.761, I25.768, I25.769, I25.700, I25.710, I25.791, I25.798, I25.799 | Not applicable |
| 1. Other ischemic heart disease | 414.0  414.8  414.9 | I25.10, I25.110, I25.5, I25.6, I25.701, 25.711, I25.720, I25.721, I25.728, I25.729, I25.731, I25.738, I25.739, I25.750, I25.751, I25.758, I25.759, I25.760, I25.761, I25.768, I25.769, I25.790, I25.791, I25.798, I25.799, I25.810, I25.811, I25.812, I25.89, I25.9 | Not applicable |
| 1. Aortocoronary bypass status convert | V45.81 | Z95.1 | Not applicable |
| 1. Percutaneous transluminal coronary angioplasty status | V45.82 | Z95.5, Z98.61 | Not applicable |
| 1. Coronary artery bypass surgery (CABG) | 36.10  36.11  36.12  36.13  36.14  36.15  36.16  36.17  36.19  36.03 | 0210083, 0210088, 0210089, 021008C, 021008F, 021008W, 0210093, 0210098, 0210099, 021009C, 021009F, 021009W, 02100A3, 02100A8, 02100A9, 02100AC, 02100AF, 02100AW, 02100J3, 02100J8, 02100J9, 02100JC, 02100JF, 02100JW, 02100K3, 02100K8, 02100K9, 02100KC, 02100KF, 02100KW, 02100Z3, 02100Z8, 02100Z9, 02100ZC, 02100ZF, 0210483, 0210488, 0210489, 021048C, 021048F, 021048W, 0210493, 0210498, 0210499, 021049C, 021049F, 021049W, 02104A3, 02104A8, 02104A9, 02104AC, 02104AF, 02104AW, 02104J3, 02104J8, 02104J9, 02104JC, 02104JF, 02104JW, 02104K3, 02104K8, 02104K9, 02104KC, 02104KF, 02104KW, 02104Z3, 02104Z8, 02104Z9, 02104ZC, 02104ZF, 0211083, 0211088, 0211089, 021108C, 021108F, 021108W, 0211093, 0211098, 0211099, 021109C, 021109F, 021109W, 02110A3, 02110A8, 02110A9, 02110AC, 02110AF, 02110AW, 02110J3, 02110J8, 02110J9, 02110JC, 02110JF, 02110JW, 02110K3, 02110K8, 02110K9, 02110KC, 02110KF, 02110KW, 02110Z3, 02110Z8, 02110Z9, 02110ZC, 02110ZF, 0211483, 0211488, 0211489, 021148C, 021148F, 021148W, 0211493, 0211498, 0211499, 021149C, 021149F, 021149W, 02114A3, 02114A8, 02114A9, 02114AC, 02114AF, 02114AW, 02114J3, 02114J8, 02114J9, 02114JC, 02114JF, 02114JW, 02114K3, 02114K8, 02114K9, 02114KC, 02114KF, 02114KW, 02114Z3, 02114Z8, 02114Z9, 02114ZC, 02114ZF, 0212083, 0212088, 0212089, 021208C, 021208F, 021208W, 0212093, 0212098, 0212099, 021209C, 021209F, 021209W, 02120A3, 02120A8, 02120A9, 02120AC, 02120AF, 02120AW, 02120J3, 02120J8, 02120J9, 02120JC, 02120JF, 02120JW, 02120K3, 02120K8, 02120K9, 02120KC, 02120KF, 02120KW, 02120Z3, 02120Z8, 02120Z9, 02120ZC, 02120ZF, 0212483, 0212488, 0212489, 021248C, 021248F, 021248W, 0212493, 0212498, 0212499, 021249C, 021249F, 021249W, 02124A3, 02124A8, 02124A9, 02124AC, 02124AF, 02124AW, 02124J3, 02124J8, 02124J9, 02124JC, 02124JF, 02124JW, 02124K3, 02124K8, 02124K9, 02124KC, 02124KF, 02124KW, 02124Z3, 02124Z8, 02124Z9, 02124ZC, 02124ZF, 0213083, 0213088, 0213089, 021308C, 021308F, 021308W, 0213093, 0213098, 0213099, 021309C, 021309F, 021309W, 02130A3, 02130A8, 02130A9, 02130AC, 02130AF, 02130AW, 02130J3, 02130J8, 02130J9, 02130JC, 02130JF, 02130JW, 02130K3, 02130K8, 02130K9, 02130KC, 02130KF, 02130KW, 02130Z3, 02130Z8, 02130Z9, 02130ZC, 02130ZF, 0213483, 0213488, 0213489, 021348C, 021348F, 021348W, 0213493, 0213498, 0213499, 021349C, 021349F, 021349W, 02134A3, 02134A8, 02134A9, 02134AC, 02134AF, 02134AW, 02134J3, 02134J8, 02134J9, 02134JC, 02134JF, 02134JW, 02134K3, 02134K8, 02134K9, 02134KC, 02134KF, 02134KW, 02134Z3, 02134Z8, 02134Z9, 02134ZC, 02134ZF, 0270046, 0270056, 027005Z, 0270066, 027006Z, 0270076, 027007Z, 02700D6, 02700DZ, 02700E6, 02700EZ, 02700F6, 02700FZ, 02700G6, 02700GZ, 02700T6, 02700TZ, 02700Z6, 02700ZZ, 0271046, 027104Z, 0271056, 027105Z, 0271066, 027106Z, 0271076, 027107Z, 02710D6, 02710DZ, 02710E6, 02710EZ, 02710F6, 02710FZ, 02710G6, 02710GZ, 02710T6, 02710TZ, 02710Z6, 02710ZZ, 0272046, 027204Z, 0272056, 027205Z, 0272066, 027206Z, 0272076, 027207Z, 02720D6, 02720DZ, 02720E6, 02720EZ, 02720F6, 02720FZ, 02720G6, 02720GZ, 02720T6, 02720TZ, 02720Z6, 02720ZZ, 0273046, 027304Z, 0273056, 027305Z, 0273066, 027306Z, 0273076, 027307Z, 02730D6, 02730DZ, 02730E6, 02730EZ, 02730F6, 02730FZ, 02730G6, 02730GZ, 02730T6, 02730TZ, 02730Z6, 02730ZZ, 02C00Z6, 02C00ZZ, 02C10Z6, 02C10ZZ, 02C20Z6, 02C20ZZ, 02C30Z6, 02C30ZZ | 33510, 33511, 33512, 33513, 33514, 33515, 33516, 33517, 33518, 33519, 33520, 33521, 33522, 33523, 33525, 33530, 33533, 33534, 33535, 33536 |
| 1. Percutaneous transluminal coronary angioplasty | 00.66 | 0270376, 02703D6, 02703E6, 02703EZ, 02703F6, 02703FZ, 02703G6, 02703GZ, 02703T6, 02703TZ, 02703Z6, 02703ZZ, 0270446, 0270456, 027045Z, 0270466, 027046Z, 0270476, 027047Z, 02704D6, 02704DZ, 02704E6, 02704EZ, 02704F6, 02704FZ, 02704G6, 02704GZ, 02704T6, 02704TZ, 02704Z6, 02704ZZ, 0271376, 02713D6, 02713DZ, 02713E6, 02713EZ, 02713F6, 02713FZ, 02713G6, 02713GZ, 02713T6, 02713TZ, 02713Z6, 02713ZZ, 0271446, 027144Z, 0271456, 027145Z, 0271466, 027146Z, 0271476, 027147Z, 02714D6, 02714DZ, 02714E6, 02714EZ, 02714F6, 02714FZ, 02714G6, 02714GZ, 02714T6, 02714TZ, 02714Z6, 02714ZZ, 0272346, 027235Z, 0272366, 0272376, 02723D6, 02723DZ, 02723E6, 02723EZ, 02723F6, 02723FZ, 02723G6, 02723GZ, 02723T6, 02723TZ, 02723Z6, 02723ZZ, 0272446, 027244Z, 0272456, 027245Z, 0272466, 027246Z, 0272476, 027247Z, 02724D6, 02724DZ, 02724E6, 02724EZ, 02724F6, 02724FZ, 02724G6, 02724GZ, 02724T6, 02724TZ, 02724Z6, 02724ZZ, 0273356, 027335Z, 0273366, 02733D6, 02733DZ, 02733E6, 02733EZ, 02733F6, 02733FZ, 02733G6, 02733GZ, 02733T6, 02733TZ, 02733Z6, 02733ZZ, 0273446, 027344Z, 0273456, 027345Z, 0273466, 027346Z, 0273476, 027347Z, 02734D6, 02734DZ, 02734E6, 02734EZ, 02734F6, 02734FZ, 02734G6, 02734GZ, 02734T6, 02734TZ, 02734Z6, 02734ZZ | Not applicable |
| 1. Percutaneous coronary intervention (PCI) with or without intra-coronary stenting | 36.01, 36.02, 36.05, 36.06, 36.07, 36.09 | 0270046, 027004Z, 0270056, 027005Z, 0270066, 027006Z, 0270076, 027007Z, 02700D6, 02700DZ, 02700E6, 02700EZ, 02700F6, 02700FZ, 02700G6, 02700GZ, 02700T6, 02700TZ, 0270346, 027034Z, 0270356, 027035Z, 0270366, 027036Z, 0270376, 027037Z, 02703D6, 02703DZ, 02703E6, 02703EZ, 02703F6, 02703FZ, 02703G6, 02703GZ, 02703T6, 02703TZ, 0270446, 027044Z, 0270456, 027045Z, 0270466, 027046Z, 0270476, 027047Z, 02704D6, 02704DZ, 02704E6, 02704EZ, 02704F6, 02704FZ, 02704G6, 02704GZ, 02704T6, 02704TZ, 0271046, 027104Z, 0271056, 027105Z, 0271066, 027106Z, 0271076, 027107Z, 02710D6, 02710DZ, 02710E6, 02710EZ, 02710F6, 02710FZ, 02710G6, 02710GZ, 02710T6, 02710TZ, 0271346, 027134Z, 0271356, 027135Z, 0271366, 027136Z, 0271376, 027137Z, 02713D6, 02713E6, 02713F6, 02713FZ, 02713G6, 02713GZ, 02713T6, 02713TZ, 0271446, 027144Z, 0271456, 027145Z, 0271466, 027146Z, 0271476, 027147Z, 02714D6, 02714DZ, 02714E6, 02714EZ, 02714F6, 02714FZ, 02714G6, 02714GZ, 02714T6, 02714TZ, 0272046, 027204Z, 0272056, 027205Z, 0272066, 027206Z, 0272076, 027207Z, 02720D6, 02720DZ, 02720E6, 02720EZ, 02720F6, 02720FZ, 02720G6, 02720GZ, 02720T6, 02720TZ, 0272346, 027234Z, 0272356, 027235Z, 0272366, 027236Z, 0272376, 027237Z, 02723D6, 02723DZ, 02723E6, 02723EZ, 02723FZ, 02723G6, 02723GZ, 02723T6, 02723TZ, 0272446, 027244Z, 0272456, 027245Z, 0272466, 027246Z, 0272476, 027247Z, 02724D6, 02724DZ, 02724E6, 02724EZ, 02724F6, 02724FZ, 02724G6, 02724GZ, 02724T6, 02724TZ, 0273046, 027304Z, 0273056, 027305Z, 0273066, 027306Z, 0273076, 027307Z, 02730D6, 02730DZ, 02730E6, 02730EZ, 02730F6, 02730FZ, 02730G6, 02730GZ, 02730T6, 02730TZ, 0273346, 027334Z, 0273356, 027335Z, 0273366, 027336Z, 0273376, 027337Z, 02733D6, 02733E6, 02733EZ, 02733F6, 02733FZ, 02733G6, 02733GZ, 02733T6, 02733TZ, 0273446, 027344Z, 0273456, 027345Z, 0273466, 027346Z, 0273476, 027347Z, 02734D6, 02734DZ, 02734E6, 02734EZ, 02734F6, 02734FZ, 02734G6, 02734GZ, 02734T6, 02734TZ, 02C03Z6, 02C03ZZ, 02C04Z6, 02C04ZZ, 02C13Z6, 02C13ZZ, 02C14Z6, 02C14ZZ, 02C23Z6, 02C23ZZ, 02C24Z6, 02C24ZZ, 02C33Z6, 02C33ZZ, 02C34Z6, 02C34ZZ | 92973, 92980, 92981, 92982, 92984, 92995, 92996 |
| **Stroke, including TIA** |  |  |  |
| 1. Transient Ischemic Attack (TIA) | 435.X | G45.0, G45.1, G45.2, G45.8, G45.9, G46.0, G46.1, G46.2, I67.841, I67.848 | Not applicable |
| 1. Acute Ischemic stroke | 433.01  433.11  433.21  433.31  433.81  433.91  434.01  434.11  434.91  436 | I63.00, I63.02, I63.6, I63.8, I63.09, I63.9, I63.10, I63.011, I63.012, I63.12, I63.013, I63.019, I63.19, I63.20, I63.22, I63.29, I63.30, I63.031, I63.032, I63.033, I63.039, I63.39, I63.40, I63.49, I63.50, I63.59, I63.111, I63.112, I63.113, I63.119, I63.131, I63.132, I63.133, I63.139, I63.211, I63.212, I63.213, I63.219, I63.231, I63.232, I63.233, I63.239, I63.311, I63.312, I63.313, I63.319, I63.321, I63.322, I63.323, I63.329, I63.331, I63.332, I63.333, I63.339, I63.341, I63.342, I63.343, I63.349, I63.411, I63.412, I63.413, I63.419, I63.421, I63.422, I63.423, I63.429, I63.431, I63.432, I63.433, I63.439, I63.441, I63.442, I63.443, I63.449, I63.511, I63.512, I63.513, I63.519, I63.521, I63.522, I63.523, I63.529, I63.531, I63.532, I63.533, I63.539, I63.541, I63.542, I63.543, I63.549, I67.89 | Not applicable |
| 1. Subarachnoid hemorrhage | 430.X | I60.00, I60.01, I60.02, I60.2, I60.4, I60.6, I60.7, I60.8, I60.9, I60.10, I60.11, I60.12, I60.30, I60.31, I60.32, I60.50, I60.51, I60.52 | Not applicable |
| 1. Intracerebral hemorrhage | 431.X | I61.0, I61.1, I61.2, I61.3, I61.4, I61.5, I61.6, I61.8, I61.9 | Not applicable |
| 1. Retinal vascular occlusion | 362.3 | G45.3, H34.00, H34.01, H34.02, H34.03, H34.9, H34.10, H34.11, H34.12, H34.13, H34.211, H34.212, H34.213, H34.219, H34.231, H34.232, H34.233, H34.239, H34.821, H34.822, H34.823, H34.829, H34.8110, H34.8111, H34.8112, H34.8120, H34.8121, H34.8122, H34.8130, H34.8131, H34.8132, H34.8190, H34.8191, H34.8192, H34.8310, H34.8311, H34.8312, H34.8320, H34.8321, H34.8322, H34.8330, H34.8331, H34.8332, H34.8390, H34.8391, H34.8392 | Not applicable |
| 1. Other cerebrovascular disease | 437.X  438.X | G45.4, G46.3, G46.4, G46.5, G46.6, G46.7, G46.8, I67.1, I67.2, I67.4, I67.5, I67.6, I67.7, I67.9, I67.81, I67.82, I68.0, I68.2, I68.8, I69.00, I69.010, I69.10, I69.011, I69.012, I69.013, I69.014, I69.015, I69.018, I69.019, I69.020, I69.20, I69.021, I69.022, I69.023, I69.028, I69.30, I69.031, I69.032, I69.033, I69.034, I69.039, I69.041, I69.042, I69.043, I69.044, I69.049, I69.051, I69.052, I69.053, I69.054, I69.059, I69.061, I69.062, I69.063, I69.064, I69.065, I69.069, I69.80, I69.090, I69.90, I69.091, I69.092, I69.093, I69.098, I69.110, I69.111, I69.112, I69.113, I69.114, I69.115, I69.118, I69.119, I69.120, I69.121, I69.122, I69.123, I69.128, I69.131, I69.132, I69.133, I69.134, I69.139, I69.141, I69.142, I69.143, I69.144, I69.149, I69.151, I69.152, I69.153, I69.154, I69.159, I69.161, I69.162, I69.163, I69.164, I69.165, I69.169, I69.190, I69.191, I69.192, I69.193, I69.198, I69.210, I69.211, I69.212, I69.213, I69.214, I69.215, I69.218, I69.219, I69.220, I69.221, I69.222, I69.223, I69.228, I69.231, I69.232, I69.233, I69.234, I69.239, I69.241, I69.242, I69.243, I69.244, I69.249, I69.251, I69.252, I69.253, I69.254, I69.259, I69.261, I69.262, I69.263, I69.264, I69.265, I69.269, I69.290, I69.291, I69.292, I69.293, I69.298, I69.310, I69.311, I69.312, I69.313, I69.314, I69.315, I69.318, I69.319, I69.320, I69.321, I69.322, I69.323, I69.328, I69.331, I69.332, I69.333, I69.334, I69.339, I69.341, I69.342, I69.343, I69.344, I69.349, I69.351, I69.352, I69.353, I69.354, I69.359, I69.361, I69.362, I69.363, I69.364, I69.365, I69.369, I69.390, I69.391, I69.392, I69.393, I69.398, I69.810, I69.811, I69.812, I69.813, I69.814, I69.815, I69.818, I69.819, I69.820, I69.821, I69.822, I69.823, I69.828, I69.831, I69.832, I69.833, I69.834, I69.839, I69.841, I69.842, I69.843, I69.844, I69.849, I69.851, I69.852, I69.853, I69.854, I69.859, I69.861, I69.862, I69.863, I69.864, I69.865, I69.869, I69.890, I69.891, I69.892, I69.893, I69.898, I69.910, I69.911, I69.912, I69.913, I69.914, I69.915, I69.918, I69.919, I69.920, I69.921, I69.922, I69.923, I69.928, I69.931, I69.932, I69.933, I69.934, I69.939, I69.941, I69.942, I69.943, I69.944, I69.949, I69.951, I69.952, I69.953, I69.954, I69.959, I69.961, I69.962, I69.963, I69.964, I69.965, I69.969, I69.990, I69.991, I69.992, I69.993, I69.998 | Not applicable |
| **Heart Failure, including Cardiomyopathy** |  |  |  |
| 1. **Heart Failure** | 398.91, 402.01, 402.11, 402.91, 404.11, 404.13, 428.0, 428.1, 428.9  404.01, 404.03, 404.91, 404.93, 428.20, 428.21, 428.22, 428.23, 428.30, 428.31, 428.32, 428.33, 428.40, 428.41, 428.42, 428.43 | I09.81, I11.0, I13.0, I13.2, I50, I50.1, I50.2, I50.20, I50.21, I50.22, I50.23, I50.3, I50.30, I50.31, I50.32, I50.33, I50.4, I50.40, I50.41, I50.42, I50.43,  I50.8, I50.81, I50.810, I50.811, I50.812, I50.813, I50.814, I50.82, I50.83, I50.84, I50.89, I50.9, I97.13 | Not applicable |
| 1. **Cardiomyopathy** | 425.11, 425.18, 425.2, 425.4, 425.5, 425.7, 425.8, 425.9 | I42.0, I42.1, I42.2, I42.5, I42.6, I42.7, I42.8, I42.9, I43, | Not applicable |
| **Other CVD Outcomes** |  |  |  |
| **Arrhythmia** | 426.0, 426.11, 426.12, 426.13, 426.3, 426.4, 426.6, 426.7, 427.0, 427.3X, 427.4X, 427.6, 427.8X, 427.9 | I44.0, I44.1, I44.2, I44.7, I45.0, I45.5, I45.6, I45.10, I45.19, I46.2, I46.8, I46.9, I47.1, I48.0, I48.1, I48.2, I48.3, I48.4, I48.91, I48.92, I49.01, I49.1, I49.02, I49.2, I49.3, I49.5, I49.8, I49.9, I49.40, I49.49, R00.1 | Not applicable |
| **Venous thromboembolic disease** | 415.1  415.11  415.19  451.1  451.2  451.8, 451.81, 451.83, 451.84, 451.89  451.9  452  453.0  453.1  453.2  453.3  453.40, 453.41, 453.42  453.5, 453.51, 453.52  453.72, 453.73, 453.74, 453.75, 453.76, 453.77, 453.79  453.8, 453.82, 453.83, 453.84, 453.85, 453.86, 453.87, 453.89  453.9 | I26.01, I26.02, I26.09, I26.90, I26.92, I26.99, I80.3, I80.8, I80.9, I80.10, I80.11, I80.12, I80.13, I80.201, I80.202, I80.203, I80.209, I80.211, I80.212, I80.213, I80.219, I80.221, I80.222, I80.223, I80.229, I80.231, I80.232, I80.233, I80.239, I80.291, I80.292, I80.293, I80.299, I82.0, I82.1, I82.90, I82.91, I82.210, I82.220, I82.221, I82.290, I82.601, I82.602, I82.603, I82.609, I82.611, I82.612, I82.613, I82.619, I82.621, I82.622, I82.623, I82.629, I82.890, I82.A11, I82.A12, I82.A13, I82.A19, I82.B11, I82.B12, I82.B13, I82.B19, I82.C11, I82.C12, I82.C13, I82.C19, T80.0XXA, T81.72XA, T81.718A, T82.817A, T82.818A | Not applicable |
| **Cardiac arrest** | 427.5 | I46.2, I46.8, I46.9 |  |
| **Myocarditis/Pericarditis** | 422.0, 422.90, 422.91, 422.92, 422.93, 422.99, 429.0, 420.0, 420.90, 420.91, 420.99 | A18.84, I30.0, I30.1, I30.8, I30.9, I32, I40.0, I40.1, I40.8, I40.9, I41, I51.4, M32.12 | Not applicable |
| **Valvular disease** | 424.0, 424.1, 424.2, 424.3  424.90, 424.91, 424.99 | A18.84, I34.0, I34.1, I34.2, I34.8, I34.9, I35.0, I35.1, I35.2, I35.8, I35.9, I36.0, I36.1, I36.2, I36.8, I36.9, I37.0, I37.1, I37.2, I37.8, I37.9, I38, I39, M32.11 | Not applicable |
| **Carotid disease** | 412.X  414.X | I25.2, I25.3, I25.41, I25.42, I25.82, I25.83, I25.84 | Not applicable |
